# Enhanced Monovision Intraocular Lenses: Current Status and Future Perspectives—Systematic Review

**DOI:** 10.3390/biomedicines14010074

**Published:** 2025-12-29

**Authors:** Zofia Honorata Trusiak, Aleksandra Leoniuk, Aleksandra Tomaszuk, Michał Sawicki, Joanna Konopińska

**Affiliations:** Department of Ophthalmology, Medical University of Bialystok, 15-089 Bialystok, Polandaleksandra.leoniuk7@gmail.com (A.L.);

**Keywords:** systematic review, cataract, monovision, intraocular lens, visual acuity

## Abstract

**Background/Objectives**: Cataract is the most common cause of blindness in the world. Enhanced monovision intraocular lenses (EMV IOLs) have been recently made available on the market. In this study, we aimed to further the understanding of EMV IOLs and their potential benefits in cataract surgery, while also identifying areas for future research. **Methods**: In this review, we discuss the findings of a few previously published comparative studies concerning different types of EMV IOLs. We conducted a systematic review of comparative studies (randomized controlled trials, prospective and retrospective observational studies) describing binocular uncorrected intermediate vision acuity (UIVA) in patients after cataract surgery and implantation of monofocal plus IOLs based on emmetropia and monovision. **Results**: The secondary outcomes measured were uncorrected distance visual acuity, uncorrected near visual acuity (UNVA; described in eight studies), spectacle independence and patients’ satisfaction. A total of 199 patients (average age 68.11 years) were analyzed in the included studies; of these patients, 169 achieved UNVA reaching an average of 0.188 logMAR. **Conclusions**: The monovision approach may provide enhanced intermediate and near vision without significantly compromising distance vision or patient satisfaction, though results varied across studies. Future randomized trials with standardized outcome measures and conducted over a longer follow-up period are warranted to confirm these findings.

## 1. Introduction

Cataract is the most common cause of blindness in the world. In Poland, approximately 350,000 cataract surgeries are performed annually, and with the growing needs of society, this number may increase to 400,000 [1]. The procedure involves a short hospitalization period and recovery time, allowing patients to quickly return to their daily duties. As the number of surgeries increases, so do the expectations of patients regarding the postoperative effect. Until now, patients in Poland have been offered several types of intraocular lenses (IOLs). Single-vision IOLs allow patients to have good visual acuity at one distance; however, this option involves the need to use glasses (usually for near distances). Multifocal IOLs with two, three, or more focal points enable good visual acuity from several distances; however, many patients have noticed undesirable effects such as “glare”, “halo”, dysphotopsia and a reduced sense of contrast postoperatively. In addition, extended depth of focus (EDOF) lenses are available in the market. These lenses have an extended focal point, which ensures a smooth range of vision with fewer undesirable effects than those associated with multifocal lenses [2]. Another new alternative to monofocal lenses are “monofocal plus” lenses such as enhanced monovision (EMV) lenses, which, thanks to many focal points along the focal axis, allow good vision acuity not only at long distances but also at intermediate distances, without the need to use corrective lenses [3]. EMV IOLs owe their efficacy to the phenomenon of spherical aberration: different focal lengths of light rays due to their position between the center and the edge of the lens [4]. EMV IOLs from various manufacturers are available on the market, including Tecnis Eyhance ICB00 (Johnson & Johnson Vision Care, Jacksonville, FL, USA), the Vivinex Impress XY1-EM (Hoya Surgical Optics, Singapore), Isopure 123 IOLs (PhysIOL, Liege, Belgium) and RayOne EMV (Rayner IOLses Ltd., West Sussex, UK)

EMV IOLs can also be implanted using the monovision, “mini” or “micro” monovision techniques. The monovision technique is based on targeting the dominant eye for distant emmetropia and the non-dominant eye for myopia of −1.25 to −2.50 diopters. “Micro” and “mini” monovision techniques target the non-dominant eye for lower myopia up to −0.75 diopter and −0.75 to −1.25 diopters, respectively [5].

EMV lenses, with their unique properties and reduced occurrence of undesirable effects such as glare or halo compared to those encountered with multifocal lenses, are gaining popularity [6]. Herein, we describe the results of a few previously published studies comparing “monofocal plus” lenses with other IOLs available on the market [6,7,8,9,10,11,12,13,14,15,16,17]. The aim of this review was to compare the outcomes of EMV IOLs implanted with two different approaches: monovision and emmetropia. This review specifically focused on analyzing binocular uncorrected visual acuity at different distances (far, intermediate and near) for both of the approaches, assessment of patient satisfaction with the two approaches, spectacle independence after implantation of these IOLs and evaluation of the effectiveness of the monovision approach in providing enhanced intermediate and near vision without compromising distance vision or patient satisfaction.

To our knowledge there are no systematic reviews with narrative synthesis that have compared these many different types of monofocal plus IOLs implanted bilaterally while dividing the patients into two groups based on the two approaches: emmetropia and monovision.

## 2. Materials and Methods

Retrospective, prospective, RCT studies conducted since 2021 that described binocular uncorrected distance visual acuity (UDVA) as well as uncorrected near visual acuity (UNVA) in patients after binocular cataract surgery and implantation of monofocal plus lenses for emmetropia and monovision were included in this review. Further, only studies with a postoperative follow-up period of at least 1 month were included. Exclusion criteria comprised studies examining patients with an axial length above 25 mm and studies in which binocular postoperative uncorrected intermediate vision acuity (UIVA) was not described. Studies published in a language other than English were also excluded.

A quantitative meta-analysis was not performed; data were synthesized narratively due to heterogeneity among study designs and outcomes.

Pooled means are unweighted arithmetic averages, not accounting for sample size, given very high heterogeneity (I^2^ > 90%); these are descriptive approximations only and should not be interpreted as meta-analytic estimates.

### 2.1. Search

PubMed, Web of Science and Google Scholar databases, clinical trials registries, conference proceedings and manufacturer websites were searched by two authors (Z.T. and J.K.) using the following terms: “enhanced monofocal” OR “monofocal plus” OR “monofocal+” OR “premium monofocal” OR “extended monofocal” OR “Tecnis Eyhance” OR ICB00 OR “RayOne EMV” OR “Vivinex Impress” OR IsoPure and “Intraocular Lenses” [MeSH] OR “Intraocular Lens Implantation” [MeSH] OR “Cataract Extraction” [MeSH] OR “Phacoemulsification” [MeSH] OR “FLACS” [MeSH] and”Visual Acuity” [MeSH] OR “Contrast Sensitivity” [MeSH] OR “Patient Satisfaction” OR “Spectacle Independence” OR “Aberrations, Optical” [MeSH] OR “Dysphotopsia”. Studies published up to June 28, 2025 were included. The last search was conducted on the 8th of December 2025. This review followed the PRISMA (Preferred Reporting Items for Systematic Review and Meta-Analysis) guidelines and was registered in the PROSPERO international prospective register of systematic reviews (PROSPERO registration number: 1106567). No separate publicly available protocol document was prepared. No protocol amendments were made after registration.

**Figure 1:**
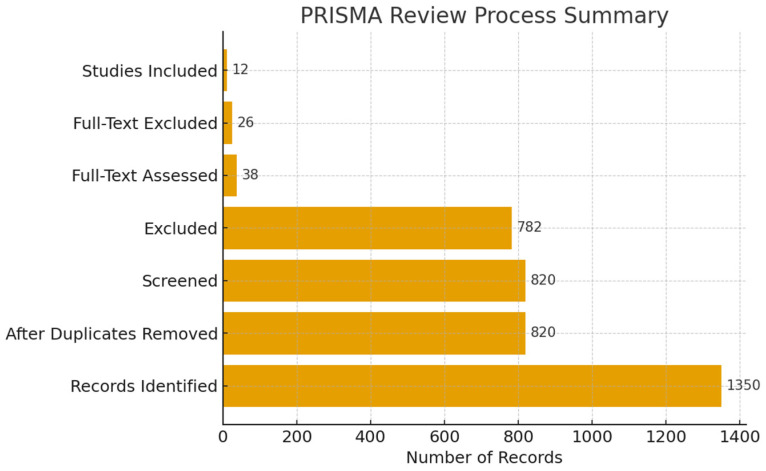
PRISMA Review Process Summary.

Potential review-level limitations include reliance on two reviewers for screening and data extraction without automation tools, which may introduce minor subjective bias. Figure 1 illustrates the PRISMA flowchart of the study selection procedure.

The PRISMA 2020 checklist can be viewed in the Supplementary Materials (Table S1).

### 2.2. Quality Assessment

Out of the 12 studies included in this analyses, five studies were progressive and the remaining five were retrospective (Table 1). Three of the five progressive studies, namely those by Donoso et al. [11], Sandoval et al. [9] and Giglio et al. [13], were randomized. Therefore, to assess the risk of bias, Version 2 of the Cochrane risk-of-bias tool for randomized trials (RoB 2) was used. The other seven studies were assessed using The ROBINS-I tool (“Risk Of Bias In Non-randomized Studies of Interventions”). All RCTs, those by Donoso et al. [11], Sandoval et al. [9] and Giglio et al. [13], were well-conducted with low or minor concerns for risk of bias. There were some missing data in the study by Sandoval, Potvin and Solomon [9], such as no clear reason given for the number of patients not completing the follow-up visits, which could subtly impact results. The prospective studies in this review were assessed to have low risk of bias across all domains. On the contrary, the retrospective studies had moderate to serious risk, primarily from potential confounding and less rigorous participant selection procedures. Table comparing the risk of bias of used studies can be viewed in Supplementary Materials (Table S2).

### 2.3. Primary and Secondary Outcomes

The primary outcome measure was binocular UIVA at 66 cm. The secondary outcome measures were UDVA 4 m and UNVA at 40 cm in 8 studies, spectacle independence and patients’ satisfaction. Data were extracted independently by two authors (Z.T. and J.K.) with a process to resolve differences.

### 2.4. Surgery

All patients included in the reviewed studies were operated upon under topical anesthesia. Overall, 270 patients underwent standard phacoemulsification procedure. Femtosecond laser-assisted cataract surgery (FLACS) was performed on 226 patients (41.71% in the monovision group and 58.85% in the emmetropia group). In most of the studies, surgeries were performed by just a few highly qualified surgeons; however, in the study by Dell et al. [6], due to the high number of patients examined, 20 experienced ophthalmic surgeons in 17 surgical centers across the UK and Ireland conducted the procedures.

### 2.5. Types of IOLs

Among the 10 included studies, five different IOLs were implanted. The most common IOL was the Tecnis Eyhance ICB00 IOL used in seven out of ten studies. Overall, 405 patients (78.89% of IOLs implanted in the monovision group and 83.50% IOLs implanted in the emmetropia group) received ICB00 IOL, 30 patients received Alcon Clareon Vivity IOL, 37 received Rayner RayOne EMV IOLs, 12 received Hoya Vivinex Impress IOLs and 12 received PhysIOL IsoPure (Table 2a,b). Technical features of the lenses used are described in Table 3. The term “monofocal plus” is still not universally standardized “Monofocal-plus” lenses combine features of monofocal and EDOF optics and some authors include them in the EDOF group instead. That said, types of IOLs used in the studies are classified as monofocal plus by the producers or authors of these studies.

## 3. Results

Twelve studies describing the performance of EMV lenses postoperatively were included in the analysis. Seven studies described the performance of monofocal plus lenses after their implantation using the monovision principle. A total of 250 patients were analyzed, with an average age of 68.11 years. UDVA in patients implanted with IOLs targeted for monovision revealed an average of 0.041 logMAR and UIVA revealed an average of 0.09 logMAR. Among the 250 patients, 169 achieved UNVA reaching an average of 0.188 logMAR. Targeted refraction for the non-dominant eye among the analyzed 199 patients reached −0.803 D (Table 4).

Eight studies examining 364 patients with an average age of 72.23 years with IOLs implanted for emmetropia revealed an average UDVA of 0.017 logMAR and UIVA of 0.165 logMAR. For the 272 patients who were tested postoperatively for near visual acuity, UNVA was 0.399 logMAR (Table 5).

The emmetropia group revealed better mean distance vision (UDVA), whereas the monovision group revealed significantly better intermediate (UIVA) and near vision (UNVA) (Table 6).

### 3.1. Satisfaction and Spectacle Independence

Satisfactory rate was similarly estimated in all the studies included. It was mostly based on questionnaires. In the study conducted by Park et al. [3], 100% of the examined patients stated that they were satisfied with the postoperative outcomes. In the monovision group, 92% patients reported that they would recommend the procedure to their families, while this percentage was 96% in the emmetropia group.

Beltraminelli et al. [4] confirmed the previously reported findings that the mini-monovision technique shows great spectacle independence in far, intermediate and near vision and higher patient satisfaction. Similarly, in the Sarcone study, the patients reported complete spectacle independence in most daily activities. Can and Bayhan [10] reported that 83.3% of the operated patients achieved total spectacle independence.

Dell et al. [6] reported that 90.6% of the patients were satisfied with the procedure and 93.2% of the patients would recommend the procedure to their families. In the study by Sandoval [9] et al., 92% of patients in the monovision group were completely or mostly satisfied with their distant vision, and 62% were satisfied with near vision without glasses. Llovet-Rausell et al. [16] reported that 97.8% of patients were either satisfied or fairly satisfied with their current vision, and 95.8% expressed that their vision at night is the same or better than before the surgery.

Similar results could be observed in the studies examining patients after bilateral IOL implantation targeted for emmetropia. Overall, 92% of the patients in the study by Garcia-Bella et al. [12] reported that their vision did not cause any problems in their everyday life, whereas 58% claimed to have no difficulty with reading text in a newspaper. Similarly, in the study by Donoso et al. [11], 97% of patients were satisfied and 70% very satisfied with their vision. The emmetropia group in the study by Sandoval et al. [9] reported satisfaction with their distant vision (94%) and near vision (41%). Similarly, in the emmetropia group in the study by Dell et al. [6], overall satisfaction was reported by 96% of the patients, and 100% of the patients said that they would recommend the procedure to their families.

### 3.2. Side Effects

In a study comparing Tecnis Eyhance in the mini-monovision and emmetropia setting, the most frequent side effect of implanted IOLs was halos [6]. In the study by Park et al. [6], 8% of patients (4 out of 50) reported discomfort due to halos three months after surgery. No patients reported discomfort due to glare or starbursts in either of the groups. In another study comparing the same IOL, there were no side effects observed. The study by Dell et al. [6] found that the most common side effect or visual phenomenon reported for the Tecnis Eyhance ICB00 IOL was glare, with 14.4% of patients experiencing moderate difficulty and 1.6% experiencing significant difficulty. Halos were reported as causing moderate difficulty in 7.6% of patients and significant difficulty in 1.8% of patients. Starburst effects led to moderate difficulty in 10.4% of patients and significant difficulty in 0.8% of patients. Further, ghosting/double vision was the least reported side effect, with 4.4% patients experiencing moderate difficulty and 0.8% experiencing significant difficulty. Authors found no statistically significant difference in the incidence of these visual phenomena between the use of Tecnis Eyhance ICB00 and the conventional Tecnis Monofocal ZCB00 IOLs. Sandoval et al. [9] found that the main side effects or visual disturbances reported with the TECNIS Eyhance were halos and sensitivity to light. However, it is important to note that these visual disturbances were not particularly bothersome to most patients. The majority of patients in both the emmetropia and monovision groups reported being “never” or “rarely” bothered by light sensitivity and halos. In a study by Scarfone et al. [7], no specific complications or adverse events for patients receiving the Clareon Vivity IOL were demonstrated. However, the authors did mention a low rate of complications associated with this treatment. Can et al. [10] reported minor side effects in the group in which RayOne EMV IOL was implanted using the mini-monovision technique. The most common side effect was dysphotopsia (glare), which occurred in 4.1% of patients (one out of twenty-four reported glare in one eye) at three and six months post-operation. One out of 12 patients noticed and was uncomfortable with a difference in refraction between the eyes. This patient had a postoperative refraction of −1.00 D in the non-dominant eye. Two patients (16.7%) required glasses, one for distance vision and one for near vision. The spectacle independence rate was 83.3%. Garcia-Bella et al. (12) reported the most common side effect for the RayOne EMV to be cystoid macular edema. Their study identified three cases of CME as non-serious adverse events. One case occurred monocularly (in one eye), while the other two cases affected both eyes of the same patient. Two of these cases demonstrated improvement before the one-month follow-up visit. The third case showed persistent visual acuity loss at the one-month and three-month visits, suggesting unresolved CME at three-month post-operation. However, the authors did not enclose any information about dysphotopsia. In a study by Donoso et al. [11] comparing enhanced monofocal IOL (ICB00) with a conventional aspheric monofocal IOL (ZCB00) implant, the most common side effects or adverse effects associated with the enhanced IOL were dysphotopic phenomena, particularly glare and halos. More than 80% of the patients in both groups reported experiencing occasional glare, halos or star flashes. However, more than 90% of the patients in both groups reported that these symptoms were mild. Overall, the study found no statistically significant differences in these phenomena between the enhanced and conventional monofocal IOL groups. In a study by Menucci et al. [14] comparing Tecnis Eyhance ICB00 Vivinex Impress XY1-EM and IsoPure 123, there were no common side effects reported for any of the IOLs studied. In fact, the study found excellent results, with no significant adverse effects, for all three enhanced monofocal IOLs examined. Specifically, the study reported that there was a total absence of halo and glare perception in all three IOL groups at three months post-operation. In a study by Giglio et al. [13] comparing Tecnis Eyhance and Clareon Vivity IOLs, authors only briefly mentioned optical side effects like glare and halos; the side effects were not the primary focus of their study (Table 7).

### 3.3. Defocus Curves

**Figure 2:**
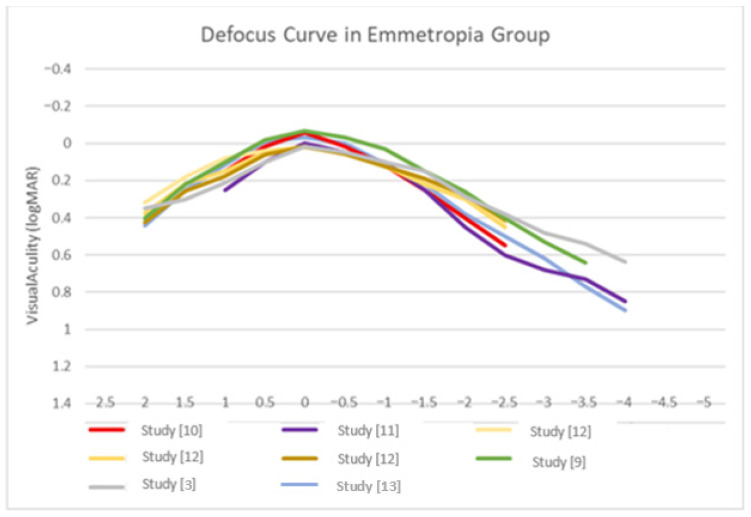
Defocus curve in emmetropia group. Data derived from published studies [3,9,10,11,12].

**Figure 3:**
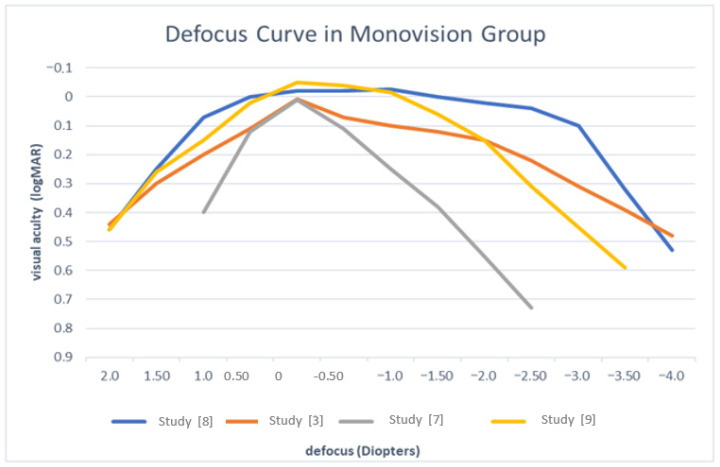
Defocus curve in monovision group. Data derived from published studies [3,7,8,9].

Figure 2 and Figure 3 illustrate the estimated defocus curves for the patients implanted with lenses in the emmetropia and the monovision groups.

### 3.4. Femtosecond Laser-Assisted Cataract Surgery (FLACS)

Two of the studies used in this article (Park et al. [3], Dell et al [6].) were based on patients undergoing femtosecond laser-assisted cataract surgery (FLACS). This method relies on the precision of short, high-power pulses at infrared wavelength (1053 nm) to perform corneal incisions, capsulotomy and lens fragmentation. Although in theory this method allows the surgeon to achieve reproducibility, it is not considered to improve visual postoperative outcomes compared to standard phacoemulsification procedure according to the database of the European Registry of Quality Outcomes for Cataract and Refractive Surgery [18].

### 3.5. Contrast Sensitivity and Wavefront Aberrations

Due to different methods used to assess contrast sensitivity in the studies it was not manageable to truly evaluate and compare the results.

Any deviation from the perfectly shaped flat wavefront at the retina is called a wavefront aberration. They can be grouped into Lower-Order Aberrations (LOA) and Higher-Order Aberrations (HOA). LOAs represent symmetrical lower complexity distortions usually corrected by glasses. LASIK or IOLs reflect basic optical power errors not optical quality. Meanwhile, HOAs represent complex and irregular distortions of the wavefront that cannot be corrected with spherical or cylindrical lenses. Only a few of the studies used contained an analysis of the wavefront aberrations with sufficient detail. The comparison of the aberrations measured can be seen in the table below (Table 8).

### 3.6. Heterogeneity Analysis

From the 12 studies used, 10 were assessed for heterogeneity. Can et al. [8] and Beltraminelli et al. [4] could not be evaluated because of missing data like SDs or numerical means.

The DerSimonian–Laird random-effects model shows very high heterogeneity across studies (I^2^ > 90%), mirroring meaningful differences in lens design, refractive targeting and testing methods. Regardless of this variability, all studies indicate that enhanced monofocal IOLs offer a broad range of functional vision. The analysis results can be seen in Table 9.

## 4. Discussion

Several contemporary premium IOLs have been introduced in the global market to enlarge spectacle independence and minimize the optical adverse events associated with multifocal IOLs. Megiddo-Barnir and Alió [2] subdivided the currently available EDOF IOLs into five categories: (1) IOLs that use spherical aberration to increase the depth of focus, (2) pinhole IOLs, (3) refractive or diffractive multifocal IOLs with low near addition, (4) ‘hybrid’ EDOF-multifocal IOLs with modest near addition and (5) IOLs with modified central optical profile that use variations in the geometry of the central optic to create an EDOF effect. In our review, outcomes of patients implanted with different types of IOLs from the fifth category, including IOLs such as Tecnis Eyhance ICB00, Alcon Clareon Vivity, Rayner RayOne EMV, Hoya Vivinex Impress and PhysIOL IsoPure in different settings (monovision or emmetropia), were compared. The review included 496 patients with a mean of age 70.75 years who underwent bilateral monofocal plus IOL implantation and compared refraction targeting emmetropia and monovision. There were 199 patients with implanted IOLs for monovision and 297 patients with implanted IOLs targeting emmetropia. Both groups, with patients of comparable ages, were examined to assess binocular visual acuity for different distances (UDVA, UIVA, UNVA) in similar conditions, patients’ satisfaction and spectacle independence. Since the studies included in this systematic review with narrative synthesis used different methods to evaluate contrast sensitivity in patients postoperatively, we were unable to compare the results reliably. Most of the studies that we analyzed focused on differentiating between IOL types and their accomplishments (e.g., EDoF, Trifocal, Toric); therefore, contrast sensitivity was another comparative measure to assess the pros and cons of different IOLs. In this study we only compared the difference between targeted refraction of the same type of IOLs (monofocal plus); contrast sensitivity was not studied as a part of this review as we did not expect it to change due to the refractive aim. The aim of this study was to prove the thesis statement that monofocal plus lenses when implanted for monovision allow patients to achieve significantly better binocular UIVA without compromising comfort of everyday life.

Our findings suggest that the monovision approach may provide enhanced intermediate and near vision without significantly compromising distance visual acuity or patient satisfaction. Risk of bias across most domains was assessed as low to moderate; however, some retrospective studies [6,7] were more prone to selection bias and confounding. Despite these limitations, the data support the use of the monovision approach with monofocal plus IOLs as an attainable option for enhancing spectacle independence. Future randomized trials with standardized outcome measures and longer a follow-up are warranted to confirm these findings and allow for a clear guidance regarding clinical recommendations.

Regarding the cost-effectiveness of implanted IOLs, to the best of our knowledge, none of the published studies have investigated the cost-effectiveness of EMV IOLs. Hu et al. [3] conducted a cost-effectiveness analysis comparing multifocal IOLs to monofocal IOLs in cataract surgery. They employed a Markov model to simulate patient outcomes and measured cost-effectiveness from both societal and healthcare sector perspectives. The analysis considered factors such as spectacle dependence, glare, haloes and the possibility of IOL exchange. The analysis found that multifocal IOLs are cost-effective from both societal and healthcare perspectives. The base case analysis found that multifocal IOLs were associated with a 0.71 quality-adjusted life year (QALY) increase at an additional cost of USD 3,415, resulting in an incremental cost-effectiveness ratio of USD 4,805 per QALY. This is well below the standard willingness-to-pay threshold of USD 50,000 per QALY. The model was most sensitive to patient age, probability of spectacle dependence and the disutility of wearing glasses. This suggests that despite their higher initial cost, multifocal IOLs provide good value for money in terms of improved quality of life. However, the cost-effectiveness of multifocal IOLs decreases with patient age, which implies that younger patients may benefit more from multifocal IOLs in terms of cost-effectiveness. Furthermore, the probability of spectacle dependence significantly affects the cost-effectiveness of multifocal IOLs. Authors suggest that improvements in IOL technology that further reduces spectacle dependence could enhance their cost-effectiveness. The study highlighted the need for more research on newer IOL technologies and their cost-effectiveness.

Lin and Yang also analyzed the cost-effectiveness of premium IOLs [4]. They analyzed the cost-effectiveness of monofocal and multifocal IOLs for cataract patients in Taiwan. Both monofocal and multifocal IOLs showed beneficial effects on postoperative vision performance and quality of life. However, multifocal IOLs demonstrated a higher rate of spectacle independence (around 90%) than did monofocal IOLs (31.8%), which suggests that multifocal IOLs could significantly improve patients’ quality of life by reducing dependence on glasses. Moreover, the study found that it costs an additional USD 57–58 to achieve an increase of 1% in the rate of spectacle-independence with multifocal IOLs. While this represents an additional cost, it may be justified by the improved quality of life for patients.

There are also some limitations regarding the monovision method of implanting IOLs. Monovision is said to limit the binocular visual acuity, with some patients experiencing side effects such as halos, reflections, haze and flashes. Furthermore, monovision influences depth perception, cortical adaptation and spatial vision. Since blurred and clear images are being processed at a different speed, it is said that monovision could cause an effect called “illusion of movement”, which causes patients to falsely perceive the distance of moving objects [19].

Our study has some limitations. First, most of studies reviewed had a follow-up period of only 1–6 months. This short follow-up duration may not capture potential long-term complications or changes in visual outcomes. Moreover, we included both standard phacoemulsification and FLACS techniques in our analysis. This variability could introduce confounding factors when comparing outcomes. Additionally, there is lack of standardization in outcome measures of the included studies, which limits the ability to draw robust conclusions about contrast sensitivity and UNVA, which was only reported for 169 out of the 199 patients in the monovision group and 242 out of the 297 patients in the emmetropia group. Despite these limitations, our study provides valuable insights into the efficacy of the monovision approach versus that of the emmetropia approach using monofocal plus IOLs.

## 5. Conclusions

After reviewing the data from all 12 studies included in this review, our findings suggest the statement thesis that monofocal plus lenses for monovision provide patients with wider range of visual acuity at intermediate distances without any losses in uncorrected distant visual acuity than do the same type of lenses targeted for emmetropia. There were no significant differences between the groups regarding postoperative patient satisfaction. The monovision approach may enhance intermediate and near vision without significantly compromising distance visual acuity or patient satisfaction, although the results varied across studies. The available evidence indicates that the use of the monovision approach with monofocal plus IOLs is a viable option for enhancing spectacle independence. These findings have implications for patients, healthcare providers and policymakers in making informed decisions about IOL choices in cataract surgery, considering both clinical outcomes and economic factors. However, there is a need for additional future randomized trials with standardized outcome measures and longer follow-up periods to confirm these findings and provide clearer clinical recommendations.

## Figures and Tables

**Table 1:** The design of the analyzed studies.

Study (First Author, Year)	Study Design Type
Park et al., 2022 [3]	Retrospective study
Beltraminelli et al., 2023 [4]	Retrospective study
Dell et al., 2024 [6]	Retrospective study
Scarfone et al., 2025 [7]	Single-center, prospective study, nonrandomized study
Can and Bayhan, 2024 [8]	Retrospective observational study
Sandoval et al., 2023 [9]	Prospective Randomized Clinical Trial (RCT)
García-Bella et al., 2024 [10]	Prospective, monocentric, noncomparative study
Donoso et al., 2023 [11]	Prospective Randomized Clinical Trial (RCT)
Mencucci et al., 2023 [12]	Retrospective comparative study
Llovet-Rausell et al., 2025 [16]	Prospective, non-comparative
Auffarth et al., 2021 [17]	Prospective Randomized Clinical Trial (RCT)
Giglio et al., 2024 [13]	Prospective randomized controlled study (single-masked)

**Table 2:** Studies including patients with IOLs implanted (**a**) for monovision and (**b**) for emmetropia.

(**a**) **Studies Including Patients with IOLs Implanted for Monovision.**			
**No.**	**Study/Author**	**Patients** ***n***	**IOL Type**
1	Park et al. [3]	25	Tecnis Eyhance ICB00
2	Beltraminelli et al. [4]	37	Tecnis Eyhance ICB00
3	Dell et al. [6]	58	Tecnis Eyhance ICB00
4	Scarfone et al. [7]	30	Alcon Clareon SY60WF
5	Can and Bayhan [8]	12	Rayner RayOne EMV
6	Sandoval, Potvin and Solomon [9]	37	Tecnis Eyhance ICB00
7	Llovet-Rausell at al [16]	51	Rayner RayOne EMV
		Total = 250	
(**b**) Studies including patients with IOLs implanted for emmetropia.			
**No.**	**Study/Author**	**Patients** ***n***	**IOL Type**
1	García-Bella et al. [10]	25	Rayner RayOne EMV
2	Donoso et al. [11]	29	Tecnis Eyhance ICB00
3	Mencucci et al. [12]	12	Tecnis Eyhance ICB00
		12	Hoya Vivinex Impress
		12	PhysIOL IsoPure
4	Sandoval, Potvin and Solomon [9]	34	Tecnis Eyhance ICB00
5	Giglio et al. [13]	30	Tecnis Eyhance ICB00
6	Dell et al. [6] (replication)	118	Tecnis Eyhance ICB00
7	Park et al. [3] (rep.)	25	Tecnis Eyhance ICB00
8	Auffarth et al. [17]	67	Tecnis Eyhance ICB00
		Total = 364	

**Table 3:** The characteristic of analyzed IOLs.

Lens Name	Material	Asphericity	UV/Blue Light Filter	Positioning	Optic Diameter	Overall Diameter	Optic Shape
RAYONE^®^ EMV	Single-piece Rayacryl hydrophilic acrylic	Anterior aspheric surface, posterior surface aspheric or spheric depending on dioptric power.	Benzophenone UV absorbing agent	Bag	6.00 mm	12.50 mm	Biconvex (positive powers)
TECNIS Eyhance IOL model ICB00	Hydrophobic acrylic	Spherical posterior surface and a modified aspheric anterior surface.	UV/Blue light filter	Bag	6.00 mm	13.00 mm	Biconvex
AcrySof^®^ IQ Vivity	Hydrophobic acrylic	Aspheric anterior surface with the wavefronting technology and spherical posteriori surface.	UV and Blue light filtering hydrophobic Copolymer	Bag	6.00 mm	13.00 mm	Biconvex
Vivinex™ IOLs	Hydrophobic acrylic	Aspheric design with square, thin and textured optic edge.	UV filter (Model XC1-SP), UV and blue light filter (Model XY1-SP)	Bag	6.00 mm	13.00 mm	Biconvex
PhysIOL^®^ ISOPURE	GFY Hydrophobic Acrylic	Anterior and posterior aspheric surfaces with high-order aspheric terms	UV and blue light filter	Bag	10 D to 24.5 D: 6.00 mm—25 D to 30 D: 5.75 mm	10 D to 24.5 D: 11.00 mm—25 D to 30 D: 10.75 mm	Aspheric biconvex surface

**Table 4:** Studies targeting patients’ refraction for monovision; post-operation results.

	Study/Author	Patients *n*	Age	Binocular UDVA	Binocular UIVA	Binocular UNVA	Duration (Months)	Refractive Aim (NDE)
1	Ella SeoYeon Park [3]	25	71.92 ± 9.98	0.10 ± 0.11	0.12 ± 0.09	0.06 ± 0.06	3	−0.95 ± 0.19
2	Tim Beltraminelli [4]	37	73.24 ± 11.3	0.10	0.10	0.35	3	−0.85 ± 0.08
3	Steven Dell [6]	58	60.07 ± 8.39	0.03 ± 0.08	0.09 ± 0.12	0.32 ± 0.16	1	−0.69 ± 0.22
4	Hugo A. Scarfone [7]	30	67.3 ± 7.25	0.01 ± 0.05	0.20 ± 0.06	-	3	−0.87 ± 0.25
5	Izzet Can [8]	12	65.75 ± 9.98	0.004 ± 0.03	0.00 ± 0.02	0.01 ± 0.02	6	−0.70 D
6	Sandoval et al. [9]	37	70 ± 4	0.00 ± 0.05	0.03 ± 0.06	0.20 ± 0.06	3	−0.76 ± 0.10 D
7	Llovet-Rausell et al. [16]	51	72.7	0.06 ± 0.09	0.25 ± 0.12	0.30 ± 0.11	3	–0.86 ± 0.33 D

**Table 5:** Studies targeting patients’ refraction for emmetropia; post-operation results.

	Study/Author	Patients *n*	Age	Binocular UDVA	Binocular UIVA	Binocular UNVA	Duration
1	Javier García-Bella [10]	25	69.2 ± 8.1	0.01 ± 0.08	0.13 ± 0.07	-	3 months
2	Donoso, Rodrigo MD [11]	29	71 ± 6	0.06 ± 0.11	0.37 ± 0.12	0.58 ± 0.15	3 months
3	Rosa Giglio [13]	30	75.10 ± 3.26	− 0.03 ± 0.07	0.17 ± 0.12	DCNVA	3 months
4	Dell (replication) [6]	118	60.07 ± 8.39	–0.08 ± 0.06	0.18 ± 0.16	0.43 ± 0.18	1 month
5	Ella SeoYeon Park [3] (replication)	25	72.2 ± 5.43	0.07 ± 0.11	0.15 ± 0.09	0.33 ± 0.13	3 months
6	Mencucci [12]						
→		12	79.38	0.03 ± 0.04	0.142 ± 0.065	0.337 ± 0.042	3 months
→		12	78.04	0.02 ± 0.04	0.133 ± 0.048	0.363 ± 0.071	3 months
→		12	78.04	0.03 ± 0.07	0.158 ± 0.097	0.354 ± 0.072	3 months
7	Sandoval et al. [9]	34	70 ± 6	00.3 ± 0.05	0.15 ± 0.06	0.40 ± 0.08	3 months
8	Auffarth et al. [17]	67	69.3 ± 8.7	0.03 ± 0.12	0.07 ± 0.12	-	6 months

**Table 6:** Comparison of both groups; post-operation results.

Group	Patients *n*	Mean Age (Years)	Mean UDVA	Mean UIVA	Mean UNVA
Emmetropia	364	72.23	0.017 logMAR	0.165 logMAR	0.399 logMAR (in 272 patients)
Monovision	250	68.79	0.0557 logMAR	0.1271 logMAR	0.1943 logMAR (in 220 patients)

**Table 7:** Side effects of studied IOLs.

Study	IOL(s) Studied	Reported Side Effects	Frequency/Details	Key Conclusions
Park et al. [3]	Tecnis Eyhance (mini-monovision vs. emmetropia)	Halos	8% (4/50) reported discomfort at 3 months	No glare or starbursts; overall low dysphotopsia
Dell et al. [6]	Tecnis Eyhance ICB00 vs. Tecnis Monofocal ZCB00	Glare	14.4% moderate; 1.6% significant	No statistical difference vs. monofocal
		Halos	7.6% moderate; 1.8% significant	-
		Starbursts	10.4% moderate; 0.8% significant	-
		Ghosting/diplopia	4.4% moderate; 0.8% significant	Rare; lowest reported phenomenon
Sandoval et al. [9]	Tecnis Eyhance (emmetropia vs. monovision)	Halos, light sensitivity	Mostly “never” or “rarely” bothersome	Symptoms were mild, not impactful
Scarfone et al. [7]	Clareon Vivity (enhanced monofocal/EDOF-like)	None significant	Low complication rate overall	No dysphotopsias described
Can et al. [8]	RayOne EMV (mini-monovision)	Glare	4.1% (1 eye of 24) at 3–6 months	Mild, infrequent glare
		Anisometropia symptoms	1 patient uncomfortable with −1.00 D target	Monovision sometimes symptomatic
		Spectacle dependence	16.7% required glasses	Spectacle independence: 83.3%
García-Bella et al. [10]	RayOne EMV	Cystoid macular edema (CME)	3 non-serious cases; 2 resolved quickly	No data on glare/halos
Donoso et al. [11]	Tecnis Eyhance ICB00 vs. ZCB00	Glare, halos, star flashes	>80% reported occasional symptoms, >90% reported mild intensity	No difference between IOL types
Mencucci et al. [12]	Tecnis Eyhance, Vivinex Impress, IsoPure	None reported	Complete absence of halos/glare at 3 months	All three enhanced monofocals performed extremely well
Giglio et al. [13]	Tecnis Eyhance vs. Clareon Vivity	Brief mention of halos/glare only	Not quantified; not primary endpoint	Not enough data for comparison
Llovet-Rausell et al. [16]	RayOne EMV	None reported	-	No asthenopia symptoms were reported and contrast sensitivity was maintained

**Table 8:** Comparison of measured aberrations.

Study/Year	IOL Types Compared	Aberrations Measured	Main Findings on Aberrations	Overall Interpretation
Mencucci et al., 2023 [12] (J. Clin. Med.)	Tecnis Eyhance, Vivinex Impress, IsoPure	Total aberrations, HOAs, LOAs, spherical aberration, coma, trefoil, OSI, PSF	No significant differences across the three enhanced monofocal IOLs. HOAs and spherical aberration remained low. OSI/PSF indicated high optical quality.	Enhanced monofocals do not introduce clinically meaningful aberrations.
Park et al., 2022 [3] (Sci. Rep.)	Enhanced monofocal mini-monovision	Not measured	High patient satisfaction + maintained contrast sensitivity → indirectly suggests low levels of HOAs.	Optical quality preserved; aberrations likely similar to monofocals.
Beltraminelli et al., 2023 [4] (BMC Ophthalmol.)	Enhanced vs. standard monofocal (mini-monovision)	Not measured	No deterioration in contrast sensitivity → indicates minimal induced aberrations.	Enhanced monofocal preserves monofocal-like aberration profile.
Dell et al., 2024 [6] (Clin. Ophthalmol.)	Enhanced vs. standard monofocal	Not measured	Equivalent contrast sensitivity; no increase in dysphotopsias.	Supports no increase in HOAs or SA.
Sandoval et al., 2023 [9] (Clin. Ophthalmol.)	Enhanced monofocal (emmetropia vs. monovision)	Not measured	No loss of optical quality; stable contrast sensitivity.	Aberrations likely unchanged between targets.
García-Bella et al., 2024 [10] (JCRS)	Bilateral enhanced monofocal IOL	Not measured	Smooth defocus curve without photic effects.	Consistent with low spherical aberration and HOAs.
Donoso et al., 2023 [11] (JCRS)	Enhanced vs. standard monofocal	Not measured	No contrast sensitivity penalty; similar dysphotopsia profile.	Suggests similar internal aberrations.
Can and Bayhan, 2024 [8] (Turk. J. Ophthalmol.)	Enhanced mono-EDOF vs. trifocal	Not measured	Trifocal had slightly reduced optical quality; enhanced monofocal maintained clean PSF clinically.	EM lenses have much lower HOAs than multifocals.
Scarfone et al., 2025 [7] (Life)	New enhanced monofocal targeted for mini-monovision	Not measured	Maintained contrast and image quality.	Aberration profile remains monofocal-like.
Llovet-Rausell et al. [16]	Enhanced monofocal targeted for mini-monovision	Not measured	Contrast sensitivity maintained, satisfaction with night vision.	Optical quality was evaluated only indirectly.

**Table 9:** Heterogeneity analysis of ten assessed studies.

Outcome	k (Arms)	Pooled Mean (logMAR)	95% CI	Cochran’s Q	I^2^ (%)
UDVA (Uncorrected Distance VA)	10	0.016	−0.022 to 0.054	236.3	96.2%
UIVA (Uncorrected Intermediate VA)	10	0.164	0.125 to 0.203	136.0	93.4%
UNVA (Uncorrected Near VA)	7	0.397	0.348 to 0.446	81.0	92.6%

## Data Availability

All materials and information are available upon an e-mail request to the corresponding author.

## Supplementary Materials

The following supporting information can be downloaded at: https://www.mdpi.com/article/10.3390/biomedicines14010074/s1. Table S1: PRISMA 2020 Checklist; Table S2: Risk of Bias Assessment of Included Studies.

## Abbreviations

The following abbreviations are used in this manuscript:

EMV	enhanced monovision
IOL	intraocular lens
RCT	randomized controlled trial
UIVA	uncorrected intermediate visual acuity
UDVA	uncorrected distance visual acuity
UNVA	uncorrected near visual acuity
EDOF	extended depth of focus
PRISMA	Preferred Reporting Items for Systematic Review and Meta-Analysis
FLACS	femtosecond laser assisted cataract surgery
QALY	quality-adjusted life year
